# Randomized, controlled trial to analyze the effect of using a traction-bed-device on patients suffering from osteoarthritis/spondylosis of the lumbar spine

**DOI:** 10.1186/s12891-025-08961-w

**Published:** 2025-08-23

**Authors:** Jörn Bengt Seeger, Friedemann Müller

**Affiliations:** 1 Kurpark Klinik Bad Nauheim, Kurstraße 41-45, Bad Nauheim, 61231 Germany; 2 Department of Orthopaedics and Orthopaedic Surgery, University Hospital Giessen and Marburg (UKGM), Giessen, Germany; 3 Schön Klinik Bad Aibling, Kolbermoorer Straße 72, Bad Aibling, 83043 Germany

**Keywords:** Traction, Spondylosis, Back pain, Disability evaluation, Quality of life

## Abstract

**Background:**

Treatment methods for low back pain (LBP) can be divided into conservative, invasive and surgical treatment approaches with traction therapy as a non-surgical therapeutic option. A clinical study analysed the effect of using a traction-bed-device (Movento) on 35 patients suffering from osteoarthritis/spondylosis of the lumbar spine.

**Methods:**

The study was performed as a multicentric, double-blind, randomised, controlled interventional study. The patients were treated over three weeks while staying in rehabilitation clinics. All patients were assessed initially at study entry, weekly and after 3 weeks as well as 12 weeks after discharge. The following outcome measures were used: Numerical Rating Scale (NRS), Roland-Morris Disability Questionnaire (RMDQ), (12) Progressive Isoinertial Lifting Evaluation (PILE-Tests) and the 36-Item Short Form Health Survey (SF36).

One hundred ten patients between 40 and 75 years of age with a diagnosed osteochondrosis/spondylarthrosis with chronification stadium 1 and 2 according to Gerbershagen were enrolled in the study. Both study groups received conventional rehabilitation therapy. The intervention group additionally received additional therapy with a minimum of five hours with the Movento traction device per night with seven sessions per week and a duration of 21 days. The therapy is based on the unloading and loading of spinal tissueThe duration of the treatment was limited to a minimum of 5 h and a maximum of 8 h.

**Results:**

The intervention group was able to show significantly better results in pain reduction (NRS) (*p* < 0.05), the Roland-Morris Questionnaire (*p* < 0.05), the PILE-Test (*p* < 0.05), the morning start-up time and the Finger-Floor-Distance measurement (*p* < 0.05) as well as the improvements in quality of life (SF-36).

**Conclusions:**

The presented results show that an additional traction device can improve pain score, function, clinical scores as well as improvements in quality of life in patients with spondylosis.

## Key Points

**Findings:**

Therapy with the traction-bed-device (Movento) in combination with specific back pain rehabilitation achieves statistically significant results compared to treatment without this device.

**Implications:**

The results show that an additional traction device improves pain score, function, clinical scores as well as life quality and should be added to conservative rehabilitation methods.

**Caution:**

It cannot be assumed that traction is always the same. In addition the effect of a 3 weeks treatment was not be maintained at 12 weeks after cessation of the intervention

## Background

Low back pain (LBP) represents one of the most common and cost-intensive medical challenges worldwide with a lifetime prevalence of 74–85% [1]. It can be assumed that arthritis of the lumbar facet joints as well as chronic lumbalgia without radicular symptoms (Spondylarthrosis) account for 10 to 41% of specific back pain and osteochondrosis accounts for 26 to 39% of cases [2, 3].

Treatment methods can be divided in conservative, invasive and surgical treatment approaches. Whereas conservative treatments consist of physical therapy such as transcutaneous electrical nerve stimulation (TENS) and manual therapy techniques, epidural steroid injections or nerve block can be described as invasive treatments. Surgical treatment options can be utilized for severe or refractory cases. The aim of traction therapy as an adjunct treatment is to reduce intervertebral disc pressure and alleviate nerve root compression.

Current minimal invasive therapeutic options are able to relieve pain, although they need anesthetics and have potential side effects [4].

Non-Surgical Interventions are associated with small to moderate, usually short-lived effects on pain [5, 6]. One non-surgical therapeutic option is traction therapy which is applied by physiotherapists in manual therapy [7] or using a mechanical traction device (traction benches) [8–10].

The traction-bed is a therapy concept based on the principle of traction i.e. the loading and unloading of the spine and spinal tissue.

The core differentiator between the existing traction therapies and the used system is the relatively low amount of traction weight (dosage) applied over a much longer period of time (min. 5 h duration of application). As such the device is mainly developed for a home-use application where the patient is self-treating during the night.

The purpose of this study was to evaluate whether therapy with the traction-bed-device (Movento) in combination with standard inpatient rehabilitation program specific back pain recan achieve better results on pain score, function scores as well as quality of life compared to a treatment without this device in patients with spondylarthritis/osteochondrosis.

Comparative therapies (inversion therapy and intermittent traction therapy) differ significantly in terms of dosage (traction weight) and duration of application. Movento utilises the patient's body weight (in the horizontal plane) and dispenses with additional forces and is applied over several hours during the night.

## Methods

### Material

**Fig. 1:**
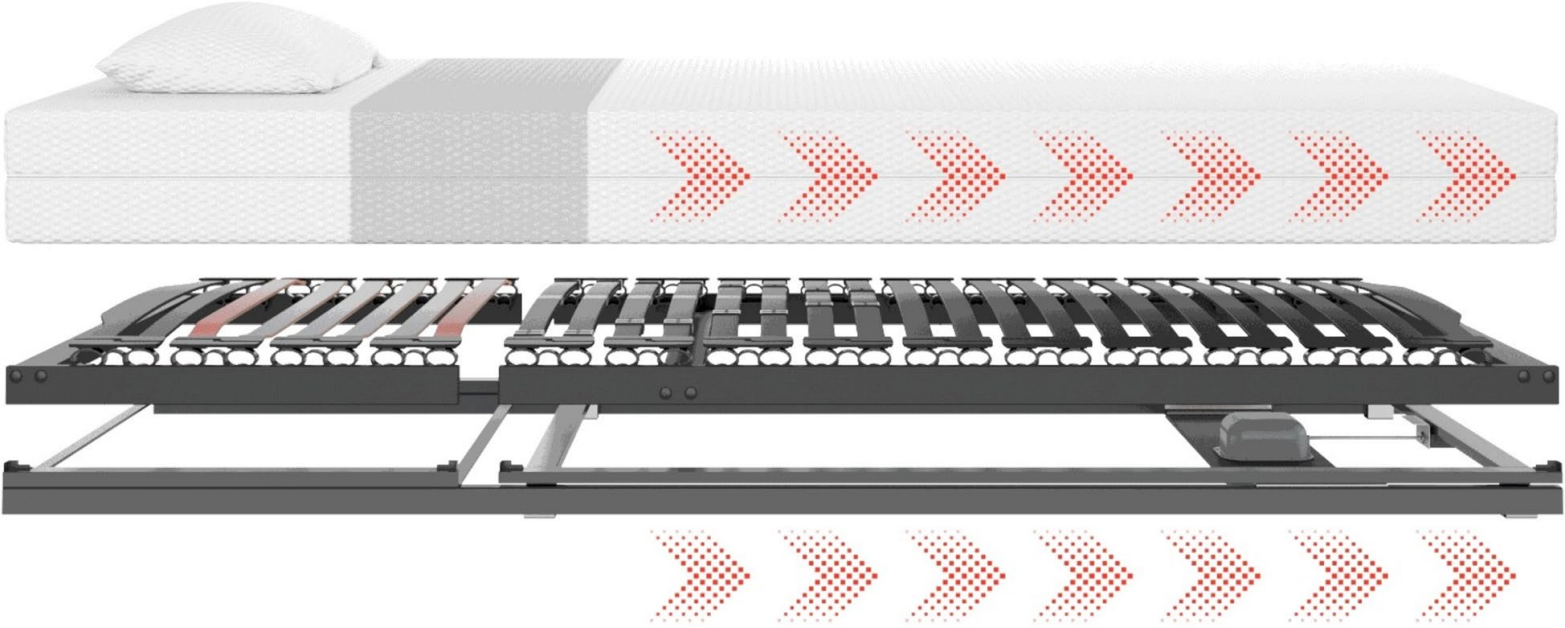
Traction-bed Movento

The traction-bed (Fig. 1) is a therapeutic device based on the principle of intermitting traction projected onto the spine and the spinal tissue. The lower part of the bed (with the pelvis and the legs) is moved along the longitudinal axis of the body by an electric motor and the upper part (with the upper part of the body, head, shoulder and arms) remains fixed. As a result, the spinal muscles are mobilized smoothly, whereby the movement is concentrated on the lumbar part of the spine. The amount of traction projected (as a function of speed and distance applied) is variable and as such adjusted to the individual need of the patient. The speed of the traction-bed could be adjusted between 0.8 and 4.0 mm/s, the hub by 1.5–6.0 cm and the running time between 1 and 8 h according to the comfort of the patient with a minimum of 5 h running time.

### Participants

Recruitment and randomization were undertaken at several orthopedic rehabilitation facilities, the Fachklinik Bad Bentheim, the Dr. Ebel Fachklinik “Moorbad” Bad Doberan, the Kurpark-Klinik Bad Nauheim, the Reha-Kliniken Küppelsmühle Bad Orb, between May 2021 and January 2023. A total of 101 patients were included in the study.

Inclusion criteria: Male/female patients between 40 and 75 years of age with degenerative spine disease. The age limit was based on the expected physiological changes in this population. Body weight between 40 and 120 kg, body height between 150 and 190 cm based on the technical specifications of the device used. Indication: Lumbar spondylosis with chronification of pain (stadium 1 or 2 according to Gerbershagen). i.e. intermittent or persisting pain with potential of irradiation on neighboring body regions and inappropriate use of pain killers. The Gerbershagen classification, also known as the Mainz Pain Staging System (MPSS), is a system used to classify chronic pain patients based on the severity and chronicity of their pain.

Exclusion criteria: Disabilities above 50% according to German social security law, patients with lumbar stenosis, psychiatric disorders (e.g. depression, psychosis, dementia, alcohol or drug abuse) inflammatory conditionsn (e.g. discitis, myositis), rheumatic flare, osteoporosis (to avoid traction induced fracture), scoliosis, radicular symptoms, tumors, open wounds, or pregnancy.

Written informed consent was obtained from every patient. The study was approved by the ethics committee of the Bayerischen Landesärztekammer, Mühlbaurstr.16 D-81677 München and registered on 16/12/2019 under the registration number DE/EKBY10/00055992 in accordance with the Declaration of Helsinki. Clinical trial registration number is DRKS00025086 and date of registration was 16/04/2021.

### Procedures

A total of 101 participants were concluded in the study with 64 female and 37 male patients.

**Fig. 2:**
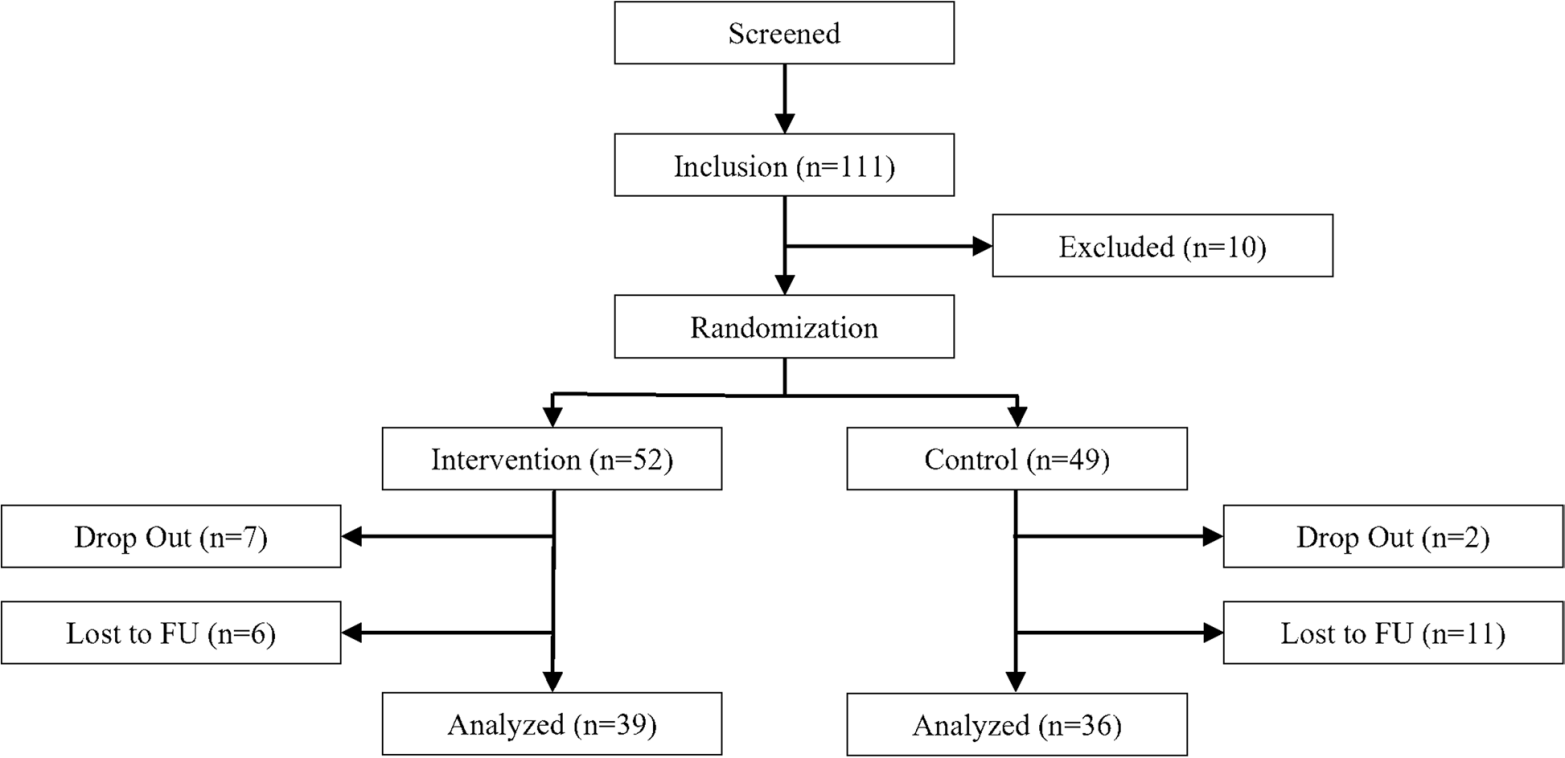
Clinical trial flowchart

Participant demographics are summarized in Table 1, and the enrollment and allocation process is illustrated in the CONSORT flow diagram (Fig. 2).

**Table 1:** Demographics (n = number, M = Mean, SD = Standard deviation)

	Total		Intervention		Control	
	**n**	**%**	**n**	**%**	**n**	**%**
Sex						
Male	64	63.37%	34	65.38%	30	61.22%
Female	37	36.63%	18	34.62%	19	38.78%
	**M**	**SD**	**M**	**SD**	**M**	**SD**
Age	55.30	6.077	55.63	5.61	54.94	6.57

The study was carried out as a randomised, controlled clinical study with a parallel group study design. The patients as well as the examiners were blinded to the treatment allocation Randomization was carried out by the online-tool Urbaniak, G. C., &Plous, S. (2013) Research Randomizer (version 4.0). The patients were randomized by the rooms to which they were allocated, half of the rooms were equipped with a functional traction-bed (traction being projected) and the other half of the rooms had a non-functional (no traction being projected, mock) traction-bed (cluster randomization). This was to avoid unblinding errors by a logistic need to exchange the devices.

Primary outcome parameter of the study was the reduction of pain during the three-weeks stay in the rehabilitation clinic. The pain has been classified according to the numeric rating scale (NRS) in values from 0 (no pain) to 10 (strongest imaginable pain).

μ0 is the expected reduction in pain of the control group after three weeks of treatment on the day of discharge and μ1 representing the reduction of pain of the intervention group. Therefore, null hypothesis H0 is that the reduction of pain of the intervention group is not different from the reduction of pain of the control group (μ0 = μ1). Due to the pilot character of the study to be validated with the one-sided 2-samples t-test for mean values with α = 0,05.

Both study groups received the same amount of conventional rehabilitation therapy (according to the rehabilitation therapy standards of the German pension insurance fund) that included movement therapy, functional and work-related therapies, massage, disease-related patient training, psychological interventions, pain management, nutritional therapy above others [11]. The intervention group received the additive treatment of traction for at least 5 h in an active state and patients could adjust the range and speed of the traction movement before going to bed so that it is comfortable for them with a speed between 0.8–4.0 mm/s and a hub of 1.5–6.0 cm with a maximum traction force of the body’s own weight of the buttocks and lower extremities resting on the moving part of the mattress.

This treatment was carried out for 21 consecutive days during the stay of the patient in the rehabilitation clinic.

All patients were assessed by blinded observers initially at study entry, weekly and after 3 weeks (t0, t7, t20). The outcome measures used were Numerical Rating Scale (NRS), Roland-Morris Disability Questionnaire (RMDQ) [12], Progressive Isoinertial Lifting Evaluation (PILE-Tests), the reduction of morning start-up time as well as the finger-floor-distance measurement and quality of life measured by the 36-Item Short Form Health Survey (SF36) [13].

The pain has been classified according to the numeric rating scale (NRS) in values from 0 (no pain) to 10 (strongest imaginable pain). The Roland-Morris Disability Questionnaire (RMDQ) was used to assess self-rated physical disability caused by low back pain [14]. The PILE-Test was incorporated to assess the ability to perform repetitive lifting as quickly as possible and the SF36 for the objective measure of the quality of life [13]. In the PILE-Test the lifting capacity can be assessed by evaluating upper body flexion and lifting weights by adding 2.5 kg in each repetition until pain level has been achieved.

12 weeks after discharge the follow-up was performed by a standardized assessment with repeating the NRS, SF-36 as well as the start-up time in the morning and Finger-Floor Distance (FFD).

### Statistics

The study was carried out as a randomised, controlled clinical study with a parallel group study design. Examiner as well as patients were blinded.

Primary outcome parameter of the study was the reduction of pain during the three-weeks stay in the rehabilitation clinic.

μ0 is the expected reduction in pain of the control group after three weeks of treatment on the day of discharge and μ1 representing the reduction of pain of the intervention group.

- H₀: μ₁ ≤ μ₀
- H₁: μ₁ > μ₀

Statistical analyses were performed with the one-sided 2-samples t-Test for mean values with α = 0,05.

### Evaluation population and missing data

Due to the pilot nature of the study, it was analyzed using the Per-Protocol (PP) population. An additional Intent-to-Treat (ITT) population was not planned. Any analysis of treatment discontinuations was defined in the statistical analysis plan. No imputation of missing values was performed because of the small proportion of missing data and the potential for introducing bias through imputation.

### Calculation of population size

Based on a pilot study, the estimated standard deviation was assumed to be 2.33, resulting in an effect size of 0.5 which is supposed to be clinically significant. Using a one-sided 2-sample t-test for means with a significance level of 5% and a power of 80%, it was determined that 50 patients per treatment arm were needed to conclude a statistically significant statement. The calculation was carried out with G*power 3.1.4.9.

## Results

### Primary outcome

The subjectively rated pain values as primary endpoint show a treatment effect in both treatment arms indicating a reduction in reported pain by the standard of care.

**Fig. 3:**
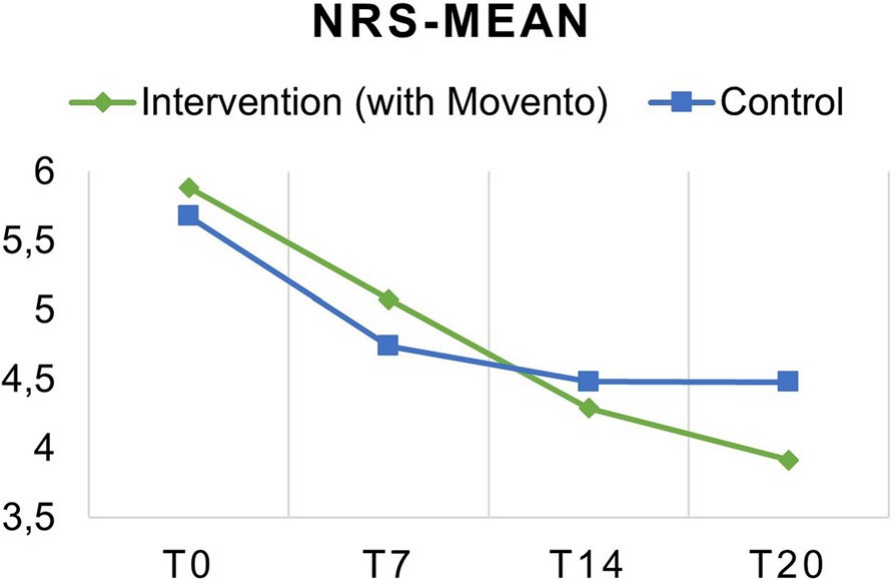
Primary Outcome: NRS-Mean of both groups over 3 weeks (from T0 until T20). The pain reduction in the intervention group (with Movento– System) was significantly superior to that in the control group

The evaluation of effectiveness is based on the data regarding the primary endpoint. In Tables 2 and 3, the subjectively collected pain values are summarized for each assessment time point Fig. 3, as well as the change from each assessment time point compared to baseline (t0) using descriptive statistics. The descriptive analysis of the individual assessment time points and the change in pain values shows a treatment effect in both treatment arms, indicated by a reduction in reported pain*.*

**Table 2:** subjectively collected pain values (NRS) are summarized for each assessment time point from t0 until t20 and the follow-up

		Count	Mean	SD
NRS t0	**Intervention**	52	5.88	2.05
	**Control**	49	5.68	1.77
NRS t7	**Intervention**	51	5.07	2.32
	**Control**	49	4.74	2.32
NRS t14	**Intervention**	49	4.29	2.06
	**Control**	48	4.48	2.50
NRS t20	**Intervention**	45	3.92	1.98
	**Control**	47	4.48	2.53
NRS Follow-up	**Intervention**	39	4.30	2.05
	**Control**	36	4.01	2.35

**Table 3:** Changes of subjectively collected pain values (NRS) between assessment time points

		Count	Mean	SD	*p*-value
NRS t0 to t7	**Intervention**	51	0.86	1.58	0.051753
	**Control**	49	0.93	2.28	0.045935
NRS t0 to t14	**Intervention**	49	1.70	2.07	0.000178
	**Control**	48	1.19	2.18	0.011399
NRS t0 to t20	**Intervention**	45	1.96	1.71	0.000007
	**Control**	47	1.17	2.07	0.011663
NRS t0 to Follow-up	**Intervention**	39	1.41	1.92	0.000453
	**Control**	36	1.46	2.49	0.001188

The positive change in pain values at all assessment time points is attributable to the applied standard therapy (Table 4).

**Table 4:** Analysis of variance of changes in NRS in both groups

	**Intervention**			**Control**			
	T-Value	*p*-Value	Decision (α = 0,05)	T-Value	*p*-Value	Decision (α = 0,05)	Significance
t0 to t7	1.9553	0.051753	n.s	2.0072	0.045935	H0 rejected	*P* < 0.05
t0 to t14	3.8103	0.000178	H0 rejected	2.5513	0.011399	H0 rejected	*P* < 0.05
t0 to t20	4.6003	0.000007	H0 rejected	2.5431	0.011663	H0 rejected	*P* < 0.05
t0 to follow-up	3.5583	0.000453	H0 rejected	3.284	0.001188	H0 rejected	*P* < 0.01

The partial eta-squared (η^2^) indicates a statistically significant (*p* = 0.01379) effect of medium size for the entire evaluation population of the control group.

The t-test analysis aligns with the previously observed trends (Table 5). For the time periods t0 to t14 and t0 to t20, positive effects of pain reduction are observed in the intervention group compared to the control group, which are also evident in the descriptive statistics. The effect of the intervention group is most pronounced at the t20 time point, as indicated by the increasing statistic values and decreasing *p*-values, demonstrating significance for this change and meeting the superiority margin of 0.1, based on the chosen significance level and it was considered meaningful by experts. Therefore, at the time of discharge, the pain reduction in the intervention group can be considered significantly superior to that in the control group.

**Table 5:** t-test change in NRS from t0 to the assessment time points

Aspin-Welch Unequal-Variance T-Test for Superiority by a Margin							
Superiority Hypothesis: (Movento = active) > (Movento = placebo) + 0,01							
	Alternative hypothesis	Mean difference	Standard error	T-statistic	DF	*p*-Value	Superiority (α = 0,05)?
t0 to t7	μT > μC + 0,01	−0.077831	0.393571	−0.2232	85.31	0.58803	No
t0 to t14	μT > μC + 0,01	0.5186225	0.4320498	1.1772	94.46	0.12103	No
t0 to t20	μT > μC + 0,1	0.7876596	0.395502	1.7387	88.16	0.04279	Yes
t0 to follow-up	μT > μC + 0,01	−0.045513	0.5170152	−0.1074	65.75	0.54259	No

### Secondary outcomes

#### Roland-morris disability questionnaire

The functional impairment based on pain was assessed using the Roland and Morris Disability Questionnaire—German version [12]. The questionnaire was administered to the patients both pre- and post-rehabilitation on the discharge day at the t20 visit. It was statistically shown with significance that the intervention group is significantly superior to the control group at the time of discharge (using absolute difference as assessing change) with the Aspin-Welch Unequal-Variance T-Test (*p* = 0.02734; mean differene = 1.869948).

#### PILE test

In addition, functional impairments within the physiotherapeutic assessment process were assessed using the PILE-Tests test, developed by Tom G. Mayer at the Productive Rehabilitation Institute of Dallas for Ergonomics (PRIDE) [15, 16]. The tests were conducted on the patient at baseline (t0) and on the day of discharge (t20). For the upper body it could be proven with the Aspin-Welch Unequal-Variance T-Test that the intervention group is statistically significant superior to the control group at the time of discharge (*p* = 0.01151; mean difference = 2.002364). For the lower body no statistical significance could be seen.

#### Reduction in Start-up time in the morning and Finger-Floor Distance (FFD)

The morning start-up time was recorded in the electronic case report form (eCRF) and supported by a Finger-Floor Distance (FFD) exercise. The morning start-up time was assessed four times: at visit t7, visit t14, visit t20, and during the follow-up, measurement could not be performed at t0 during admissal. The Finger-Floor Distance measurement required the involvement of healthcare professionals and was therefore only conducted at three time points, starting with visit t7 that serves as the baseline value for both aspects.

For both measurements the intervention group is significantly superior to the control group at the time of discharge (*p* = 0.03089).

#### Change in quality of life (SF36)

The improvement in quality of life was assessed using the SF-36 questionnaire on three occasions: baseline at t0 visit, at the time of discharge (end of the three-week study period) at t20, and during a follow-up telephone interview 12 weeks after discharge. Based on the physical component summary, which can be considered the crucial component in this clinical trial, a stronger improvement in score values was observed in the intervention group compared to the control group and the superiority of the intervention group in terms of quality of life was shown with the use of the T-test (*p* = 0.0624).

## Discussion

Vertical traction commonly leads to positive effects in osteochondrosis of the lumbar spine. The traction-bed-device (Movento) performs a therapy of mild traction during night time for up to 8 h. All other products use fixation and gravity as a principle. Shabaz et al. proved in their study that mechanical traction is more effective than manual therapy for relieving radicular pain in cervical spondylosis at C5-C6 [17].

Borodulina et al. examined the effect of mechanical traction and fresh water for the treatment of pain in the lower back [10]. During the treatment, motor and daily activity significantly improved according to the Oswestry scale and a significant increase in strength was noted in all muscle groups. These findings are comparable to our results however only 14 patients were enrolled in this study. In addition only 6 procedures were performed with a duration of 30 min and in our study we observed 21 procedures with a duraction of 5 to 8 h. According to the Roland-Morris questionnaire pain syndrome and its effect on the patient’s activity decreased.

### Pain reduction and functional improvements

Cheng et al. demonstrated, that compared with sham or no traction, lumbar traction exhibited significantly more pain reduction and functional improvements in the short term in patients with herniated intervertebral discs, but not in the long term [18]. This corresponds to our study results with a significant reduction of pain values in the intervention group compared to the control group at the end of the intervention. However, long-term results have not been assessed so far.

Minetto et al. proved in their study with a combination of rehabilitation therapies with a mattress topper in patients with lower back pain a pain reduction and an improvement of sleeping quality as well as mobilization of the lower back [19]. Nevertheless, they did not perform any passive or active movements while laying in bed or sleeping.

### IDD-therapy

McClure and Farris demonstrated a non-invasive therapy for spinal rehabilitation with the use of 20 physiotherapeutic sessions with a duration of over 25 to 30 min over a six-week period [20]. The so-called IDD therapy allows a controlled distraction of the spinal vertebra in order to mobilize the articulation and produce a negative pressure in the addressed intervertebral disc. It is assumed that this negative pressure stimulates the diffusion of liquids and nutritive substances in order to stimulate healing. Authors postulate that the negative pressure even causes a retraction of herniated nucleus pulposus. Compared to the traction-bed-device (Movento) the IDD-therapy is logistically very extensive (20 sessions etc.) in comparison to a movement during sleep with a long duration of at least 5 h, however similarities focusing on pain reduction and better function could be seen.

### Movento therapy

Movento is superior/predominant to the mentioned decompression methods because the method is highly reconcilable with everyday life, it can be used prophylactical as well as therapeutical and it can be used during sleep (basically without time loss by doctor's visits, special instructions, education, manipulation etc.).

Therapeutic use of traction in intervertebral disc degeneration, skoliosis, lumbar back pain and radiculopathy has been seen for years [8]. Aim of the therapy is pain reduction and restoration of mobility [8]. By using mechanical forces the pressure in the spine developed by gravity is reduced [9].

Prasad et al. showed, that by additional application of inversion-traction in 76.9% in the interventional group surgical intervention could be prevented [21].

### Limitations

With the traction-bed-device (Movento) traction is applied in a smooth, continuous way without jolt. Each single traction is of less relevance since no high tractional forces are applied to the body. The long duration (of up to 8 h over night) leads to a smooth and sustainable therapy of spine tissue. Similar studies with this duration cannot be found and it represents one unique feature of Movento.

Even with the use of a mock device (only presenting sound without movement) double-blind studies with these medical devices remain challenging. Patients still have expectations about their treatment based on information and some patients may correctly guess their treatment based on side effects or other clues. In the presented study patients mentioned, they did not feel any movements.

Shin et al. concluded that especially in weakened muscle force traction can be dangerous [22]. The uncomfortable positioning of the patients leads to short interventions of six times 2-min interventions in established traction setups [22].

Since patients do not always lay on their back during sleep it cannot be assumed that traction is always the same. But since a person changes the sleeping position up to 20 times per night, there is no danger of a one-sided therapy but it is evenly distributed. However, it can be assumed that different sleep positions may have an effect on increasing or decreasing treatment effectiveness.

## Conclusions

The study indicates that therapy with the traction-bed-device (Movento) in combination with specific back pain rehabilitation achieves statistically significant results on pain score, function, clinical scores as well as life quality compared to a treatment without this device. After cessation of the intervention the effect diminished again. Duration of 3 weeks was based on the logistics of inpatient rehabilitation. It is most likely that a more prolonged intervention and use of the technique in the home-healthcare setting will be effective, but this has not yet been studied.

## Future research directions

More flexible usage patterns should be studied to enable more patients to use this easy to apply device.

## Data Availability

Data (demographics, score results (NRS, RMDQ, PILE, SF36, pain value) are available upon request from corresponding author via email.
